# Comparative phytochemical and pharmacological analysis of two cultivars of *Annona squamosa* L. cultivated in Egypt

**DOI:** 10.1038/s41538-024-00368-6

**Published:** 2025-01-15

**Authors:** Safaa Yassin, Samah M. Elsohafy, Amr El-Hawiet, Maged S. Abdel-Kader, Doaa A. Ghareeb, Fikria A. Darwish, Masouda E. Amer

**Affiliations:** 1 Department of Pharmacognosy, Faculty of Pharmacy, Alexandria University, Alexandria, Egypt; 2 Department of Pharmacognosy, College of Pharmacy, Prince Sattam Bin Abdulaziz University, Al-Kharj, Saudi Arabia; 3 Department of Biochemistry, Biological Screening and Preclinical Trial Laboratory, Faculty of Science, Alexandria University, Alexandria, Egypt

**Keywords:** Cancer, Plant sciences, Chemistry

## Abstract

This study compared two *Annona squamosa* L. cultivars, Abdelrazik (*Annona A*.) and Balady (*Annona B*.), in terms of their chemical profile, in vitro cytotoxicity against HCT-116 and A549 cell lines, and total acetogenin. In addition, the two cultivars pulp were compared regarding carbohydrates and magnesium ions content and immunomodulating activity. The two cultivars were also differentiated genetically by DNA barcoding using the universal primer matK and the specific primer *Annona squamosa* matK. The results showed that *Annona A*. seeds had higher acetogenin content and exhibited more potent cytotoxic activity against the two cell lines. In contrast, *Annona B*. pulp had higher carbohydrate content and lower magnesium ions content. The splenic lymphocyte proliferation assay revealed that *Annona A*. pulp extract was slightly more active as an immunostimulant. The specific primer used for DNA barcoding was more effective for species identification, while the universal primer was better for cultivar differentiation. Overall, our findings indicate the potential for using active compounds of *Annona squamosa* L. cultivars to develop new therapeutic agents for cancer therapy and immune enhancement.

## Introduction

*Annona* L. is one of the most important genus of the Annonaceae^1^. This genus is native to the tropical region and is characterized by its edible fruits and medicinal value^2^. It is the second largest genus of the Annonaceae, including about 166 species^3^. *Annona cherimola*, *Annona muricata*, *Annona reticulata*, *Annona squamosa, Annona glauca*, *Annona montana*, *Annona scleroderma*, *Annona glabra*, and *Annona purpurea* are the most common species of this genus^3^. *Annona squamosa* L., known as sugar apple, is considered one the most widely distributed species of *Annona*^4^. It is a small deciduous tree, cultivated in tropical South America, southern Mexico and occasionally in southern Florida^5^. In the 17th century, it has been introduced into southern China, Queensland, Australia, Polynesia, Hawaii, tropical Africa, Egypt, and the lowlands of Palestine^5^.

The phytochemical studies showed that acetogenins, diterpenes, alkaloids, and cyclopeptides are the main constituents of *Annona squamosa* L.^6^. Reviewing the phytochemical literature of *Annona squamosa* L., acetogenins and cyclopeptides are concentrated mainly in seeds, while majority of alkaloids were isolated from the leaves, and diterpenes from bark and stem^6–8^. Acetogenins are considered the main bioactive compounds of Annonaceae^9^. Acetogenins structure, that is a derivative of long chain fatty acid, appear to have common features including long aliphatic chain with butyrolactone ring and substituted with one or more tetrahydrofuran ring, epoxide ring, hydroxyl groups or double bond^10^. Acetgenins are classified into four groups according to the substitution along the hydrocarbon chain^11^. The first group is linear acetogenins, in which the aliphatic chain is substituted with oxygenated moiety (hydroxyls, ketones, epoxides) or double bond, but not furan ring^12^. The second one is mono-tetrahydrofuran acetogenins that is substituted by one tetrahydrofuran ring, while the third group, bis-tetrahydrofuran acetogenins, is substituted by two tetrahydrofuran rings, either adjacent or non-adjacent and finally the fourth group is miscellaneous acetogenins, including acetogenins with tri-tetrahydrofuran rings or tetrahydrofuran and tetrahydropyran rings^12^. Alkaloids are the second major active constituent of *Annona squamosa* L.^13^. The majority of alkaloids were isolated mainly from the leaves, with aporphine alkaloids being the most common type of alkaloid in *Annona*^14^.

*Annona squamosa* L. is a medicinally active plant which is rich in many active constituents that can be effective for different health problems. The different parts of the plant, seeds, leaves, and fruits, contribute to the pharmacological activities which include anti-tumor, anti-inflammatory, antioxidant, anti-thyroid, antidiabetic, anti-microbial, anti-malarial, immunomodulatory, anti-ulcer, hepatoprotective, vasorelaxant, anti-atherogenic and wound healing activity^15^. Anti-tumor activity of *Annona squamosa* L. was reported for different plant organs, and against different cell lines. Both aqueous and organic extract of *Annona squamosa* L. seeds were found to induce apoptosis in breast carcinoma and erythroleukemia^16^. Alcoholic leaf extract has a potential anticancer activity against colon cancer cell line^17^, while the chloroform extract of fruit pericarp showed a specific potential activity against lymphoma cell line^18^. In addition, some animal trials showed that ethanolic bark extract may prevent the tumor formation DMBA (7,12-dimethylbenz (a) anthracene) induced hamster buccal pouch carcinogenesis^19^. *Annona squamosa* L. acetogenins show anti-cancer activity by inhibition of mitochondrial complex 1 and blocking of NADH oxidase enzyme, leading to depletion of ATP and cell death^20^.

Immunomodulatory agents are substances that affect the response of our immune system^21^. They may be immunostimulants that help the body fight infections and diseases, or immunosuppressants that may be essential in some auto-immune diseases and organ transplantation^21^. ASPW80-1, a polysaccharide isolated from *Annona squamosa* L. pulp, and its sulfated derivative, ASPW80-M1, were reported to have immunostimulant activity by enhancing the proliferation of mouse spleen lymphocytes^22^.

*Annona squamosa* L. Abdelrazik (*Annona A*.) and Balady (*Annona B*.) are two cultivars grown in Egypt^23^. Fruit size, seed count, and leaf dimensions are all distinguishing characteristics of the two cultivars. *Annona A*. fruits are characterized by their high weight and large size. It weighs about twice as much as *Annona B*. fruit and is about one-half the size of the *Annona b*. fruit^24^. On the other hand, *Annona B*. fruits have a higher number of seeds compared to *Annona A*. one^24^. In terms of leaf parameters, *Annona A*. leaf is about twice as large as the *Annona B*. leaf^24^.

As the two cultivars are marketed in Egypt with a high price difference (*Annona* A. price is about 3 times that of *Annona* B.), and it was claimed that *Annona* A. cultivar has great medicinal value for cancer patients over *Annona* B., so we aimed to compare the two cultivars in terms of their chemical profile, in vitro cytotoxicity against HCT-116 and A549 cell lines, total acetogenin of seeds extract, pulp carbohydrates and magnesium ions content and immunomodulating activity. The two cultivars were also differentiated genetically by DNA barcoding.

## Results

### TLC screening

The plate sprayed with anisaldehyde/sulfuric acid reagent showed differences between the leaves, seeds, and pericarp methylene chloride extracts of the two cultivars either regarding the presence of certain compounds or their concentration. These differences can also be detected in the anisaldehyde plate developed in a more polar system, while the pulp extracts of the two cultivars were almost the same. The plate sprayed with Kedde’s reagent revealed that the methylene chloride extract of the seeds of each cultivar is rich in acetogenins with almost similar bands in the two cultivars. The plate sprayed with dragendorff’s reagent showed the presence of alkaloids in the ethanol extract of the two cultivars leaves, with more intense bands in the *Annona B*. cultivar. Also, acetogenins of seeds extract showed a false positive result with dragendorff’s reagent as lactones containing compounds give false positive result with dragendorff’s reagent^25^.

### In vitro cytotoxicity

The cytotoxicity study was conducted for fractions containing acetogenins and alkaloids, as they were reported to be the major active metabolites of *Annona squamosa* L. with known cytotoxic activity^6,26,27^. The pulp extract was also used to detect whether the edible part contributed to the cytotoxicity of the plant, as the fruits were traditionally used as an anti-tumor agent^28^. IC_50_ values of different extracts (methylene chloride extract of seeds, ethanol extract of leaves and 50% ethanol extract of pulp) of the two cultivars indicated different potencies against A549 (lung) and HCT-116 (colon) cell line (Table 1).

**Table 1:** IC_50_ (µg/mL) of *Annona squamosa* L. cultivars different extracts

	*Annona B*.			*Annona A*.			Positive control
**Cell line**	**Seed** **(CH**_**2**_**Cl**_**2**_ **extract)**	**Leaves** **(C**_**2**_**H**_**5**_**OH extract)**	**Pulp** **(50% C**_**2**_**H**_**5**_**OH extract)**	**Seed** **(CH**_**2**_**Cl**_**2**_ **extract)**	**Leaves** **(C**_**2**_**H**_**5**_**OH extract)**	**Pulp** **(50% C**_**2**_**H**_**5**_**OH extract)**	**Cisplatin**
**A549 (lung)**	0.85 ± 0.00	2.40 ± 0.16	3.83 ± 0.44	0.58 ± 0.00	2.45 ± 0.07	5.05 ± 0.21	0.09 ± 0.00
**HCT-116 (colon)**	0.33 ± 0.02	1.24 ± 0.02	1.65 ± 0.04	0.23 ± 0.02	0.72 ± 0.05	2.84 ± 0.09	0.09 ± 0.11

All results are presented as mean of three determinations ± standard deviation.

The methylene chloride extract of *Annona A*. seeds had the most potent cytotoxic effect against A549 and HCT-116 cell lines, with IC50 values of 0.58 ± 0.00 µg/mL and 0.23 ± 0.02 µg/mL, respectively, followed by the methylene chloride extract of *Annona B*. seeds, which had IC50 values of 0.85 ± 0.00 µg/mL and 0.33 ± 0.02 µg/mL against A549 and HCT-116 cell lines, respectively. The cytotoxic effect of the two cultivars’ leaves extract against A549 cell line was almost the same, with IC_50_ value of 2.45 ± 0.07 µg/mL for *Annona* A. and 2.40 ± 0.16 µg/mL for Annona B. On the other hand, HCT-116 cell line was more sensitive to *Annona A*. leaves than to *Annona B*. leaves with IC_50_ value of 0.72 ± 0.05 µg/mL and 1.24 ± 0.02 µg/mL respectively. The pulp extract of the two cultivars showed the least efficacy of all tested extracts against the two cell lines. A549 and HCT-116 cell lines showed a higher sensitivity to *Annona B*. pulp, with IC_50_ value of 3.83 ± 0.44 µg/mL and 1.65 ± 0.04 µg/mL respectively, while *Annona A*. pulp, with IC_50_ value of 5.05 ± 0.21 µg/mL and 2.84 ± 0.09 µg/mL respectively. HCT-116 cell line is more sensitive to the two cultivars’ different extracts than A549 cell line.

### Characterization of the isolated acetogenin

Compound A is a colorless wax, that gave a positive color reaction with Kedde’s reagent, which is characteristic of acetogenins^29^. The EI-mass spectrum showed a peak at *m/z* 621 (M-H) and high-resolution mass spectrum with a peak at *m/z* 645.4701 (M+Na) pointed to acetogenins of molecular weight 622. Reviewing acetogenins literature, there are 28 acetogenins of the same molecular weight, 622. The NMR spectral data analysis (Table 2) reduced the probabilities to 24 acetogenins with a molecular formula C_37_H_66_O_7_^27,30–40^. The ^13^C-NMR showed carbon signals at δ 169.3, 133, 150.92, 78.23 and 17.9 which with ^1^H-NMR signals at 7.29 (br s), 5.1 (m) and 1.4 (d) ppm for position C-35, C-36, and C-37, respectively, confirmed the presence of *α,β*-unsaturated-γ-lactone nucleus with an α-methyl group to the lactone ring.

**Table 2:** ^1^H-NMR and ^13^C-NMR spectral data of compound A in CD_3_OD

C	δC, ppm	δH, ppm
1	169.3	--------
2	133	--------
3	24.61	2.26 (t, J = 7.7 Hz)
4	27.21	1.5–1.57 (m)
5	29.05	Overlapped signal. 1.3–1.37 (m)
6	29.2	
7	29.26	
8	29.29–29.4	
9		
10		
11		
12		
13	25.43	
14	32.88	1.42–1.46 (m)
15	73.55	3.41 (dt)
16	82.85	3.82–3.91 (m)
17	28.87	1.66 (m) 1.99 (m)
18	28.37	
19	81.98	3.82–3.91(m)
20	81.98	
21	28.13	1.66 (m) 1.99 (m)
22	28.87	
23	82.39	3.82–3.91 (m)
24	72.44	3.62 (m)
25	33.1	1.5–1.57 (m)
26	21.77	1.67 (m)
27	37.04	1.42–1.46 (m)
28	70.94	3.55 (m)
29	37.04	1.42–1.46 (m)
30	26.02	Overlapped signal. 1.3–1.37 (m)
31	29.36	
32	31.7	
33	22.34	
34	13.09	0.92 (t, J = 6.6 Hz)
35	150.92	7.29 (br s)
36	78.23	5.1 (m)
37	17.9	1.4 (d, J = 6.8 Hz)

The presence of two adjacent tetrahydrofuran rings with two adjacent hydroxyl groups was confirmed by carbon signals at 73.55, 82.85, 81.98, 81.98, 82.39, and 72.44 ppm for position C-15, C-16, C-19, C-20, C-23 and C-24, respectively. In addition to ^13^C NMR, the furan rings with the two hydroxyl groups were also confirmed by ^1^H NMR with oxy-methine multiple proton signal at the region of 3.8 to 3.9 which assigned for carbon 16, 19, 20 and 23 while signal at 3.4 and 3.6 were assigned for carbons attached to the hydroxyl groups. The appearance of an additional carbon signal at 70.94 ppm indicated the presence of an additional hydroxyl group within the aliphatic carbon chain.

**Fig. 1:**
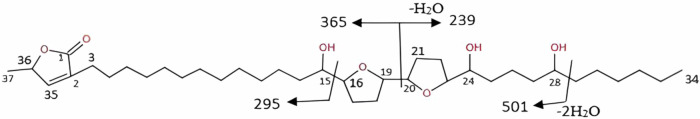
Mass fragmentation of compound A. The figure describes the different fragments detected in EI-MS of the isolated compound.

The compound EI-MS fragmentation (Fig. 1) showed an ion peak at *m/z* 501 due to the fission at C-28/C-29 followed by dehydration, while the peak at *m/z* 365 was due to the fission at C-19/C-20. The EI-MS spectra also revealed a fragment ion at *m/z* 295 due to fission at C-15/C-16, and another peak at *m/z* 239 due to fission at C-19/C-20 followed by loss of water.

**Fig. 2:**
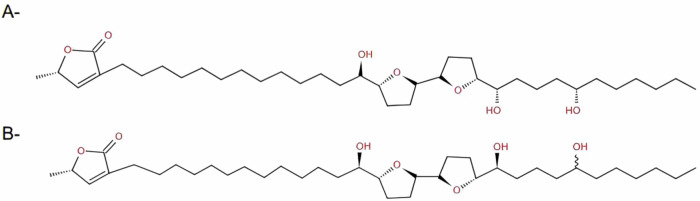
Structure of the two suspected acetogenins. Acetogenins that are structurally similar to the isolated compound, **A**- Squamocin, **B**- Squamocin D.

Combining the NMR spectral analysis and the mass fragmentation spectral data of the isolated compound, the probabilities were narrowed to only 2 acetogenins, squamocin^36^, also known as annonin I^30^, and squamocin D^35^ (Fig. 2).

**Fig. 3:**
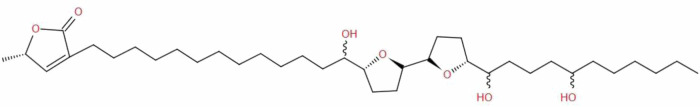
Structure of the isolated compound. The structure was detected by comparing the spectral data with the literature.

Unfortunately, we couldn’t determine the stereochemistry of the hydroxyl groups. Therefore, the isolated compound A (Fig. 3) is one of the two acetogenins, squamocin and squamocin D, or maybe a new acetogenin, and further spectral analysis is required to distinguish between them.

### Quantitation of total acetogenin of seeds extract

Acetogenins content of the seeds extract was quantified using JustTLC® software, a new generation of TLC analysis software that allows quantitative analysis on TLC plate (Sweday, Sweden, www.sweday.com). The method was validated according to ICH (International Conference on Harmonization) parameters, accuracy, precision, limit of detection, limit of quantitation, linearity, range, and specificity (Table 3)^41^.

**Table 3:** Linearity, LOD, LOQ accuracy and precision of the methods for total acetogenin quantitation

Regression equation		y = 14.603X + 21.343
Determination coefficient (R^2^)		0.99
Linearity rang in µg/band		2.1–5.6
LOD		0.31
LOQ		1.05
Precision (%RSD)	Inter-day	0.70
	Intra-days	0.94
% Recovery		99.97

*x* applied amounts (μg/band), *y* spot volume in JustTLC® software.

**Fig. 4:**
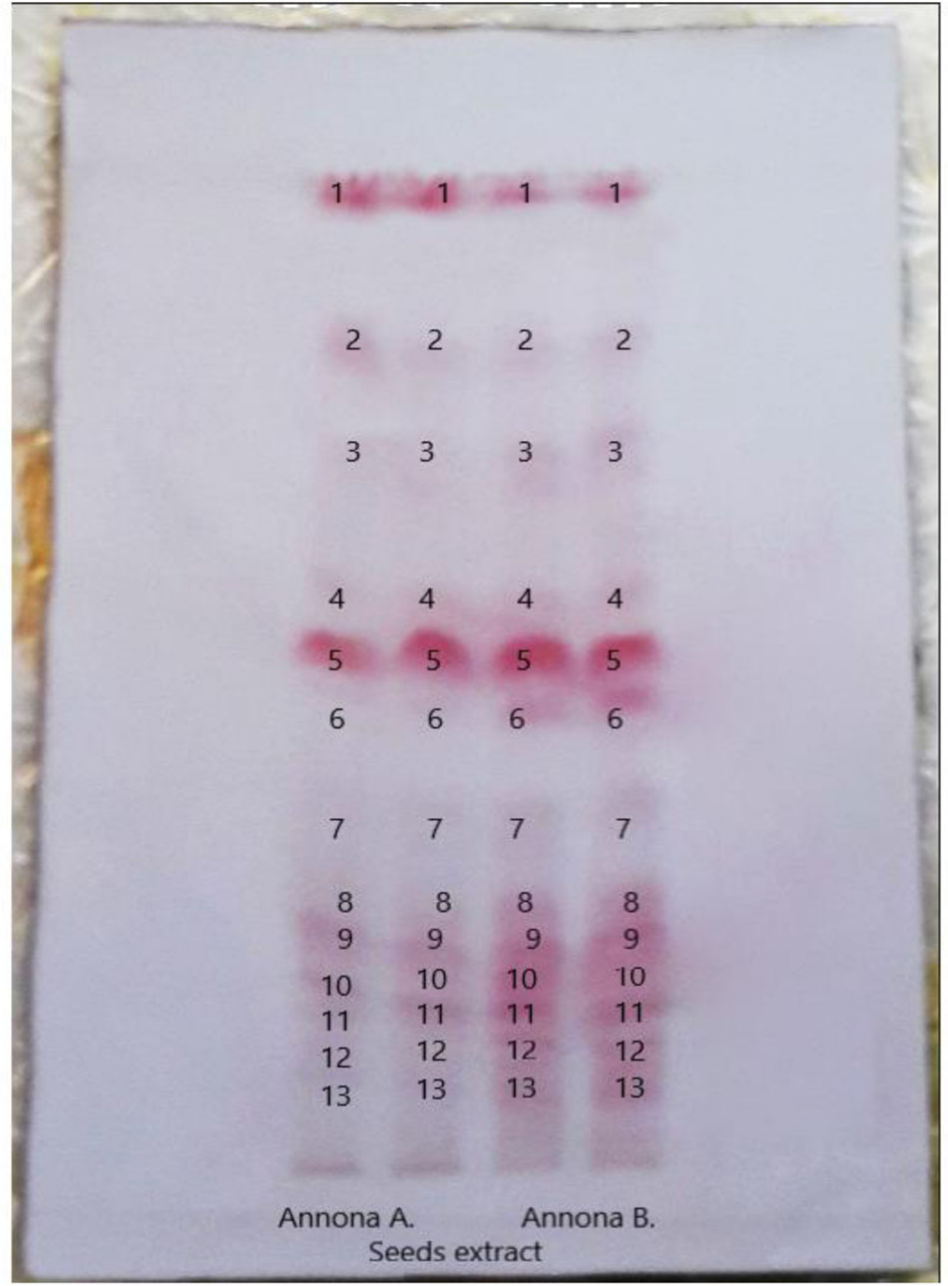
TLC plate of the two cultivars seed extracts. TLC plate of methylene chloride seed extracts of the two cultivars sprayed with Kedde’s reagent, and the volume of the spots was detected by JustTLC software.

The linear relation between the concentration of standard reference solution and spot volume was used to calculate the total acetogenin content in the sample’s solution. The findings showed that there are about 13 acetogenin bands in methylene chloride extracts of *Annona B*. and *Annona A*. seeds that are almost similar but differ in concentration (Fig. 4).

The acetogenin content of the two cultivars was illustrated in Table 4.

**Table 4:** Total acetogenin content of the two cultivars

Cultivar	*Annona B*.			*Annona A*.		
Spot number	Acetogenin content in µg per band	Acetogenin content in µg per mg of extract	Acetogenin content in µg per mg of dry powdered seed	Acetogenin content in µg per band	Acetogenin content in µg per mg of extract	Acetogenin content in µg per mg of dry powdered seed
1	29.6 ± 0.0	123.6 ± 0.1	10.3 ± 0.0	25.5 ± 1.1	106.3 ± 4.6	6.9 ± 0.3
2	6.7 ± 0.1	27.9 ± 0.5	2.3 ± 0.0	6.1 ± 0.0	25.4 ± 0.4	1.6 ± 0.0
3	1.6 ± 0.0	7.0 ± 0.0	0.6 ± 0.0	6.8 ± 0.2	28.5 ± 0.9	1.8 ± 0.0
4	3.2 ± 0.0	13.4 ± 0.0	1.1 ± 0.0	5.7 ± 0.1	23.7 ± 0.7	1.5 ± 0.0
5	47.1 ± 0.8	196.2 ± 3.5	16.5 ± 0.3	47.6 ± 1.2	198.5 ± 5.2	12.9 ± 0.3
6	3.5 ± 0.1	14.8 ± 0.4	1.2 ± 0.0	20.2 ± 0.1	84.3 ± 0.5	5.4 ± 0.0
7	1.3 ± 0.0	5.7 ± 1.6	0.5 ± 0.1	3.9 ± 0.1	16.2 ± 0.5	1.0 ± 0.0
8	0.4 ± 0.0	2.0 ± 0.0	0.2 ± 0.0	4.2 ± 0.4	17.8 ± 0.6	1.1 ± 0.1
9	5.1 ± 0.2	21.6 ± 0.9	1.8 ± 0.0	7.9 ± 1.0	33.1 ± 4.5	2.1 ± 0.2
10	3.4 ± 0.0	14.5 ± 0.2	1.2 ± 0.0	12.4 ± 0.4	51.9 ± 1.8	3.3 ± 0.1
11	7.0 ± 1.9	29.4 ± 8.1	2.4 ± 0.6	13.0 ± 1.0	54.1 ± 4.3	3.5 ± 0.2
12	2.6 ± 0.1	11.2 ± 0.6	0.9 ± 0.0	11.0 ± 0.4	46.1 ± 1.7	3.0 ± 0.1
13	1.6 ± 0.4	6.8 ± 1.6	0.5 ± 0.1	5.9 ± 0.6	24.7 ± 2.8	1.6 ± 0.1
Total	113.1 ± 1.5	474.1 ± 6.6	39.5 ± 0.5	170.2 ± 2.6	710.6 ± 10.9	45.7 ± 0.7

All results are calculated as mean ± standard deviation.

Acetogenins content per band was calculated using spot volume and regression equation, then the concentration per each mg of extract and dry powder was calculated. The results showed that the acetogenin content of *Annona A*. is higher than that of *Annona B*. with (710.6 ± 10.9 µg/mg extract) and (474.1 ± 6.6 µg/mg extract) respectively. These findings mirrored the results of the in vitro cytotoxicity screening performed in this study, as the methylene chloride extract of *Annona A*. seed is more potent than the *Annona B*. seed extract, which linked the seed extract’s cytotoxic action to the acetogenins concentration.

### Immunomodulating activity

#### Splenic lymphocyte proliferation assay

**Fig. 5:**
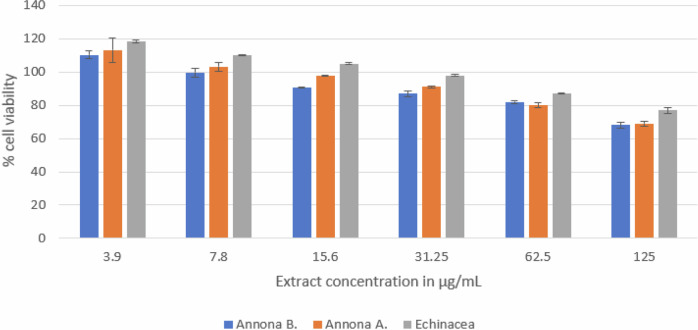
Splenic lymphocyte proliferation of *Annona A*., *Annona B* pulp extract and *Echinacea* root extract. The figure illustrates the impact of the pulp extracts from the two cultivars on the viability of spleen lymphocytes in comparison to *Echinacea* root extract. *Annona A*. showed a higher proliferative effect that decreased as the concentration of all extracts increased.

The results (Fig. 5) showed the immunostimulant activity of *Annona A*. and *Annona B*. pulp extract. Both extracts stimulated the proliferation of the spleen lymphocytes. At the same dose, *Annona A*. pulp extract showed slightly higher proliferation activity than *Annona B*. pulp extract, and their activity was very close to Echinacea extract. As the extract concentration increased <) 7.8 µg/mL), it was observed that the cells proliferation was suppressed.

#### Determination of carbohydrates content

As some *Annona squamosa* L. polysaccharides were reported to have immunostimulant effect^22^, the pulp extract total carbohydrates content was determined. The linear relation between the concentration of D-glucose solution and absorbance at 490 nm was used to calculate the total carbohydrate concentration in the sample solution. *Annona squamosa* L. pulp is rich in carbohydrates containing compounds and the findings showed that the carbohydrate content of the *Annona B*., 66.035 ± 2.249 µg/100 µg extract, is higher than that of the *Annona A*., 53.165 ± 1.664 µg/100 µg extract.

#### Quantitation of Mg^2+^ ions

The magnesium ion content of the pulp extract was detected due to its important role in the immune response^42^. It plays a role as a co-factor for immunoglobulin synthesis, immune cell adherence, C′3 convertase, antibody-dependent cytolysis, IgM lymphocyte binding, macrophage response to lymphokines and T helper–B cell adherence^43^. As EDTA forms a 1:1 complex with magnesium and calcium ions, the number of moles of ETDA consumed in the titration equals the number of moles of magnesium and calcium ions in the sample solution with EBT indicator and calcium ions only with murexide indicator.

1 $${{\rm{EDTA}}}^{4-}+{{\rm{Mg}}}^{2+}\to {[{\rm{Mg}}-{\rm{EDTA}}]}^{2{-}}$$

The results, illustrated in Table 5, showed that the concentration of magnesium ions in *Annona squamosa* L. pulp is not high enough to be a sufficient source that covers the recommended daily intake of magnesium ions, which is ~400 mg for men and 300 mg for women^44^, and that it is slightly higher in the *Annona A*. than in the *Annona B*., with 313.6 mg and 281.6 mg per kg of fresh pulp, respectively.

**Table 5:** Magnesium and calcium ions concentration per 25 mg of pulp extract calculated by complexometric titration with EDTA in each cultivar

*A. squamosa* cultivar	mmoles of Mg^2+^+Ca^2+^ ions/25 mg extract	mmoles of Ca^2+^ ions/25 mg extract	mmoles of Mg^2 +^ ions/25 mg extract	Mg^2+^ ion concentration in mg/25 mg extract	Mg^2+^ ion concentration in mg/kg fresh pulp
*Annona B.*	0.0037 ± 0.00	0.0019 ± 0.00	0.0018 ± 0.00	0.0437 ± 0.00	281.6 ± 0.00
*Annona A.*	0.0043 ± 0.00	0.0023 ± 0.00	0.0020 ± 0.00	0.0486 ± 0.00	313.6 ± 0.00

The results are presented as mean ± standard deviation.

#### DNA barcoding for cultivars differentiation

The alignment of *Annona squamosa* matK sequences for the two cultivars was efficient for species identification as *Annona squamosa* L. with percentage identity 100% obtained by 100% query cover for the *Annona B*. and with 99.04% percentage identity and 100% query cover for *Annona A*. On the other hand, this marker failed to differentiate between the two cultivars with a percentage identity of 91%.

Regarding the universal marker, matK sequences, the two cultivars were identified as *Annona squamosa* L. with lower percentage identity than the specific marker. The *Annona B*. cultivar was identified with percentage identity 95.23% and query cover of 84%, while *Annona A*. cultivar was identified with percentage identity 94.12% and a low query cover of 21%. *Annona A*. cultivar was also identified as *Annona cherimola* with the same percentage identity and query cover as *Annona squamosa*, which may confirm the reported data that *Annona A*. cultivar is a hybrid between *Annona squamosa* and *Annona cherimola*^45^. Unlike the specific primer, matK marker can be used effectively to differentiate between the two cultivars with percentage identity of 64%. In conclusion, the specific primer (*Annona squamosa* matK) is superior for species identification, while the universal (matK) is preferred for cultivars differentiation.

## Discussion

Our research findings highlight the chemical and biological differences between two *Annona squamosa* L. cultivars, Abdelrazik (*Annona* A.) and Balady (*Annona* B.). The more potent cytotoxic effect of *Annona* A. seeds extract, with a higher acetogenins concentration, seems consistent with the previous studies that indicated that acetogenins are the major cytotoxic compounds of *Annona squamosa* L.^46,47^. Acetogenins are known for their potent cytotoxicity for different cancer cell lines by enhancing cell apoptosis^48^. This supports our results that *Annona* A. showed superior cytotoxicity compared to *Annona* B.

In addition, our study supports the reported data that *Annona squamosa* L. is a rich source of nutritional elements as carbohydrates and minerals^49,50^. The analysis revealed that *Annona* B. possessed higher carbohydrate levels and lower magnesium content than *Annona* A., which emphasized the nutritional diversity of the different cultivars. The immunological study of the two cultivars showed the proliferative effect of the two cultivars pulp on the splenocytes, with a higher effect in *Annona* A. although it showed lower carbohydrate content than *Annona* B. that can be at least partially explained by the fact that the majority of carbohydrates found in *Annona* B. do not exhibit detectable proliferative activity. Furthermore, the immunostimulant activity of *Annona squamosa* L. has been reported for a single isolated polysaccharide, (ASPW80-1), not for the total carbohydrates^22^, therefore the total carbohydrate content may not be directly correlated with this activity. Our results support the promising immunostimulant effect of *Annona squamosa* L. pulp that has been described previously^51^. This also supports the traditional use of *Annona squamosa* L. as an immune-boosting agent, providing scientific evidence for its efficacy.

Furthermore, the utilization of DNA barcoding technique with both universal and specific primers provided a valuable methodology for genetic differentiation of the cultivars and for species identification that is crucial for accurate plant authentication. This information was also of benefit to prove that *Annona* A. is a hybrid cultivar. Overall, this study contributes to the scientific understanding of *Annona squamosa* L., its chemical composition, cytotoxicity, immunomodulatory properties, and cultivars differentiation, with potential implications for the development of new therapeutic agents. It also provides valuable insights for further research and in vivo studies, supported by histological tests, for the potential development of new therapeutic interventions.

## Materials and methods

### Collection of plant material

The leaves of the two cultivars of *Annona squamosa* L. were collected from El-Mamora gardens, Alexandria, Egypt, November 2019. The two cultivars’ fruits were purchased from a local market in November 2019 and identified by **Prof. Atef M. Ibrahim** professor at the Pomology Department, Faculty of Agriculture, Alexandria University. Fresh leaves of *Annona squamosa* L. cultivars, for genomic analysis, were collected from seedlings obtained from Ashraqat plant nursery in Alexandria. The experimental procedures complied with relevant institutional, national, and international guidelines and legislation, including the appropriate permission for the collection of plant specimens.

### Preparation of extracts

Fresh pulp of *Annona B*. and *Annona A*. (100 gm) was extracted with a mixture of ethanol and water (1:1) by percolation, while the dried seeds, leaves, and pericarp (30 gm) were defatted with petroleum ether, then extracted by percolation with methylene chloride, followed by extraction with ethanol. The extracts were then filtered and concentrated under reduced pressure.

### TLC screening

8 μL of the same concentration (20 mg/mL) of seeds, leaves, pericarp ethanol and methylene chloride extract together with water/ ethanol pulp extract was applied on pre-coated silica gel sheets (20 × 10) cm (E-Merck, Dramstadt, Germany) using an HPTLC applicator (Camag, linomat 5). The plates were developed in two systems of different polarity and then revealed by anisaldehyde/sulfuric acid^52^, dragendorff’s^53^ and kedde’s reagent^54^.

### In vitro cytotoxicity

The cytotoxic activity of seeds methylene chloride extract, leaves ethanol extract, and water/ ethanol pulp extract was screened against colon cancer cell line (HCT-116, ATCC CCL-247) and lung cancer cell line (A549, ATCC CCL-185), obtained from American Type Culture Collection (ATCC), using MTT assay^55^. The cells were seeded in a 96-well plate containing 100 µL of culture media per well, then incubated for 24 h The cells were then treated with different concentrations of the plant extracts, three wells for each concentration, and incubated for 48 h. After the incubation period, the cells were treated with 100 µL of fresh media containing 10 µL MTT solution (5 mg/mL) and incubated for another 3 h in CO_2_ incubator. The produced formazan crystals were dissolved using 100 µL of DMSO. Cisplatin was used as a positive control. Absorbance was measured at 570 nm against the control cells, which were treated only with culture media, using a microplate reader, and the percentage viability was calculated using the following equation:

2 $$\% {Viability}=\frac{{Absorbance\; of\; sample\; at}\,570\,{nm}}{\begin{array}{c}{Absorbance\; of\; control\; at}\,570\,{nm}\\ \end{array}}\times 100$$

The results were expressed as % viability against concentration and used to calculate IC_50_ (concentration required to kill 50% of the cells).

### Isolation of an acetogenin

The major acetogenin from *Annona B*. seeds was isolated, to be used as a standard for acetogenins quantitation, using preparative thin layer chromatography^56^. About 5 mL of 20 mg/mL solution of methylene chloride seeds extract was applied as a long band on 14 TLC plates (20 × 10) cm (E-Merck, Dramstadt, Germany). The plates were allowed to dry then developed in chloroform: methanol (19:1) system. After development, a part of the targeted zone was identified using Kedde’s reagent, then separated from the plates. The desired compound was recovered from the sorbent silica by mixture of methanol and chloroform. The resulting extract was dried to obtain about 20 mg of the target compound A, which was identified by NMR and mass spectral analysis.

### Quantitation of total acetogenin of seeds extract

The acetogenin content of *Annona B*. and *Annona A*. seeds extract was compared using image analysis program (JustTLC®) for TLC analysis (Sweday, Sweden, www.sweday.com). The previously isolated acetogenin in this study was used as a standard for conduction of the calibration curve, taking into consideration its purity detected by HPLC. The standard curve was performed by application of 3, 4, 5, 6, 7 and 8 µL of standard solution, prepared as 1 mg/mL of methanol, on pre-coated silica gel sheets (20 × 10) cm (E-Merck, Dramstadt, Germany) using an HPTLC applicator (Camag, linomat 5). For sample plate, 12 μL of each sample (20 mg/mL) was applied, together with 8 μL of the standard solution, on pre-coated silica gel sheets (11 × 20) cm. The plates were developed in chloroform: methanol (19:1) system (double run), then sprayed with Kedde’s reagent and analyzed by justTLC software.

### Validation of method

According to ICH (International Conference on Harmonization), the analytical method should be validated through evaluation of some characteristics, which are accuracy, precision, limit of detection, limit of quantitation, linearity, range, and specificity^41^. The accuracy is the measurement of how much the detected values are close to the true or reference values and can be estimated by the average percentage recovery of three identical spots of the same concentration. The accuracy was calculated by detecting the percentage of the reference recovered after analysis of three identical bands of the sample spiked with 8 µg of the reference, using the following equation:

$$\begin{array}{l}\%\, {\rm{Recovery}}=\left({\rm{Concentration}}\,{\rm{of}}\,{\rm{reference}}\,{\rm{in}}\,{\rm{spiked}}\,{\rm{sample}}\right.\\\left.\qquad\qquad\qquad\,-\,{\rm{Concentration}}\,{\rm{of}}\,{\rm{reference}}\,{\rm{un}}{\hbox{-}}{\rm{spiked}}\,{\rm{sample}}\right)/\\\qquad\qquad\qquad\quad\,{\rm{Concentration}}\,{\rm{of}}\,{\rm{added}}\,{\rm{reference}}\,\times\,100\end{array}$$

The precision is used to detect the closeness of detected values to each other and is expressed as inter and intra days percentage relative standard deviation (% RSD), which was calculated for three different analyses of the same concentration in the same day and different days. The limit of detection (LOD) is the least concentration that can be detected by the method and differentiated from zero. The limit of quantitation (LOQ) is the least concentration that can be detected with reasonable accuracy and precision. LOD and LOQ can be calculated as the following:

$$\begin{array}{l}{\rm{LCD}}={3.3}\,\times\,{\left({\rm{Standard}}\,{\rm{deviation}}\,{\rm{of}}\,{\rm{response}}/\,{\rm{Slop}}\,{\rm{of}}\,{\rm{calibration}}\,{\rm{curve}}\right)}\\{\rm{LOQ}}={10}\,\times\,{\left({\rm{Standard}}\,{\rm{deviation}}\,{\rm{of}}\,{\rm{response}}/\,{\rm{Slop}}\,{\rm{of}}\,{\rm{calibration}}\,{\rm{curve}}\right)}\end{array}$$

Linearity is the ability of the analytical method to provide a linear relation between the detected variables and can be estimated by the regression equation and determination coefficient (R^2^). The range of the analytical method is a set of values that can maintain the method’s accuracy, precision, and linearity from the lower to the upper value and is derived from the study. The specificity of the analytical method is the ability of this method to detect the analyte in the presence of many impurities. All these parameters were calculated in Table 3.

### Splenic lymphocyte proliferation assay

MTT method was used to perform the splenic lymphocyte proliferation assay. The spleens were isolated aseptically from dead mice through cervical dislocation and washed three times with PBS buffer. To achieve a homogenous cell suspension, the spleens were crushed and forced through a steel mesh (200 mesh). After 5 min of centrifugation at 1500 rpm, a Tris–HCl–NH_4_Cl solution (pH 7.2) was used to remove red blood cells. The cell pellets were then resuspended in RPMI-1640 and diluted to 5 × 10^6^ cells/mL. The cells were seeded in a 96-well plate containing 100 µL of culture media per well, then incubated for 24 h. The cells were then treated with different concentrations of the plant extracts, three wells for each concentration, and incubated for 48 h. *Echinacea* extract was used as a positive control as it was reported to have an immunostimulant effect^57^. After the incubation period, the cells were treated with 100 µL of fresh media containing 10 µL MTT solution (5 mg/mL) and incubated for another 3 h in CO_2_ incubator. The produced formazan crystals were dissolved using 100 µL of DMSO. Absorbance was measured at 570 nm against the control cells, which were treated only with culture media, using a microplate reader, followed by percentage viability calculation.

### Determination of carbohydrates content

The carbohydrate content of the pulp of the two cultivars was compared using phenol-sulfuric acid method^58^. 10 mg of D-glucose was dissolved in distilled water and the volume was adjusted to 10 mL with distilled water to prepare 1 mg/mL of standard stock solution. 100, 200, 300, 400, 500, and 600 µL of the stock solution were transferred to a 5 mL volumetric flask and the volume was adjusted with distilled water to prepare 20, 40, 60, 80, 100, and 120 µg/mL solutions. For preparation of sample solution, 50 mg of ethanol: water (1:1) fresh pulp extract of each cultivar was dissolved in distilled water and the volume was adjusted to 10 mL with distilled water to prepare a 5 mg/mL solution. 100 µL of each sample solution was transferred to a 5 mL volumetric flask and the volume was adjusted with distilled water to prepare a 100 µg/mL solution then 1 mL of each standard and sample solution was separately transferred into a clean vial. Then 500 µL of 4% phenol was added to each vial followed by 5 min shaking. 2.5 mL of concentrated sulfuric acid was added rapidly to the vials. Then the vials were permitted to cool at room temperature for 5 min. 200 µL of each solution was transferred to a microplate and the absorbance was detected at 490 nm.

### Quantitation of Mg^2+^ ions

The magnesium ions content of the two cultivars was compared by complexometric titration using ethylenediaminetetraacetic acid (EDTA) as a titrant^59^. Eriochrome Black T (ErioT) is widely used in calcium and magnesium ions titration with EDTA^60^. Murexide indicator, a specific indicator for calcium^61^, was used to remove calcium interference. For determination of total magnesium and calcium, 10 mL of each sample solution (2.5 mg/mL) was pipetted into a conical flask, then 5 mL of ammonia buffer (pH 10) was added, and one drop of Eriochrome Black T (EBT) was used as an indicator. The samples were titrated against 0.001 M EDTA. For calcium determination, 10 mL of each sample solution (2.5 mg/mL) was pipetted into a conical flask, and the pH was adjusted to 12 by addition of 2 mL of 1 M NaOH. A pinch of murexide powder was used as an indicator. The samples were titrated against 0.001 M EDTA, and the end point was calculated.

### DNA barcoding for cultivars differentiation

DNA extraction was performed using Quick-DNA plant/seed miniprep kit from **Zymo Research**, following the manufacturer’s protocol. In a **ZR BashingBead lysis tube**, 150 mg of finely divided leaves of the two cultivars were mixed with 750 µL BashingBead buffer. The samples were processed at maximum speed for 5 min using a bead beater, and then the lysis tubes were centrifuged for 1 min at 10,000 × *g*. 400 µL of the supernatant was filtered in a collection tube using a **Zymo-Spin III F** filter, then mixed after centrifugation for 1 min at 80,000 × *g*, with 1200 µL of genomic lysis buffer. For 1 min at 10,000 × *g*, 800 µL of the mixture was centrifuged in a Zymo-Spin IICR column placed in a collection tube. Zymo-Spin IICR contains a unique silica-based matrix for DNA purification. The column was then washed with two buffer solutions, 200 µL DNA Pre-Wash buffer and 500 µL g-DNA Wash buffer, with centrifugation for 1 min at 10,000 × *g* with each buffer. After that, the column was transferred to a microcentrifuge tube of 1.5 mL and 100 µL of DNA elution buffer was added to the column matrix, and then 30 s of centrifugation at 10,000 × *g* was applied to elute the DNA. A Zymo-Spin™ III-HRC Filter was placed in a clean Collection Tube and centrifuged for 3 min at 80,000 × *g* after addition of 600 µL of Prep solution. The eluted DNA was then filtered using the prepared Zymo-Spin™ III-HRC Filter and centrifuged for 3 min at 16,000 × *g*. All steps were performed at room temperature. The eluted DNA was subjected to the following application, PCR.

### Universal and specific marker sequences

The sequences of the selected universal primer (matK) and the specific primer (*Annona squamosa* matK) were listed in Table 6^61^.

**Table 6:** Sequences of universal and specific primer used

Target gene	Primer name	Primer role	Primer sequence from 5’ to 3'
matK	3F_kim	Forward primer	CGTACAGTACTTTTGTGTTTACGAG
	1R_kim	Reversed primer	ACCCAGTCCATCTGGAAATCTTGGTTC
*Annona squamosa* matK	ASqF1	Forward primer	CCATTTCCGTTTGTTCAAAC
	ASqR1	Reversed primer	GGTAAGATTTCCATTTCTTCATC

PCR was conducted according to COSMO PCR RED Master Mix, Willowfort, protocol. The master mix components were mixed in a clean and nuclease free Eppendorf for preparation of the master mix. The PCR reagents required for each sample (Table 7) were mixed and centrifuged briefly. The mixture was then incubated in a thermocycle using the thermal profile in Table 8.

**Table 7:** PCR reagents required per sample

Reagent	Volume
COSMO PCR Master Mix	25 µL
Forward and reversed primer (20µM)	1 µL
DNA template	5 µL
Nuclease free water	To 50 µL

**Table 8:** PCR reaction condition for different primers^61^

Primer	Reaction condition
matK	95 ^**◦**^C 2 min; 35 × 95 ^**◦**^C 15 s, 52 ^**◦**^C 20 s, 72 ^**◦**^C 1 min; 72 ^**◦**^C 1 min
*Annona squamosa* matK	95 ^**◦**^C 2 min; 35 × 95 ^**◦**^C 15 s, 69 ^**◦**^C 20 s, 72 ^**◦**^C 1 min; 72 ^**◦**^C 1 min

Under the mentioned PCR conditions, the products of matK and *Annona squamosa* matK primer amplification were about 900 and 400 pb, respectively.

The PCR products were purified using Genomic DNA Clean & Concentrator ® kit from Zymo Research according to the manufacturer’s protocol. In a microcentrifuge tube, each DNA sample was mixed with five times its volume of DNA binding buffer and vortexed. After that, the mixture was transferred to Zymo-spin column, placed in a collection tube, followed by centrifugation at room temperature for 30 s at 10,000 × *g*. The column was washed several times with 200 µL DNA wash buffer, with centrifugation for 30 s at 10,000 × *g* at room temperature. The column was incubated for 1 min after addition of 6 µL of DNA elution buffer and then centrifuged at room temperature for 30 s at 16,000 × *g* for DNA elution. The purified PCR products were then mixed with gel loading buffer and loaded into agarose gel to be analyzed by electrophoresis.

Chromatogram Explorer lite version 3.2 software was used to remove the low-quality ends from the sequences obtained from (**GATC Biotech, Germany**), and then by using BioEdit Sequence Alignment Editor Version 7.2.5 software, the sequences obtained from forward and reversed primers were assembled to obtain the combined sequence with the maximum length for each primer. Using NCBI Blast tool, the sequences were aligned against each other and against NCBI database.

### Statistical analysis

Data were expressed as mean ± standard deviation of the mean by the multiple comparison one-way analysis of variance (ANOVA), using Microsoft Excel (Microsoft 365 version 2407), with probability (p)- values < 0.05 considered statistically significant.

## Data Availability

All datasets used and analysed during the current study are available from the corresponding author upon reasonable request.

## References

[1] Rabelo, S. V., Quintans, J. D. S. S., Costa, E. V., da Silva Almeida, J. R. G. & Júnior, L. J. Q. Annona species (Annonaceae) oils. *Essential oils Food Preserv. Flavor safety*, 221–229 (2016).

[2] Badrie, N. & Schauss, A. G. In *Bioactive foods in promoting health* 621–643 (Elsevier, 2010).

[3] Dahiya, R. & Dahiya, S. Natural bioeffective cyclooligopeptides from plant seeds of Annona genus. *Eur. J. Med. Chem.* **214**, 113221 (2021). 10.1016/j.ejmech.2021.113221 33540356

[4] Martínez-Maldonado, F. E., Miranda-Lasprilla, D. & Magnitskiy, S. Sugar apple (Annona squamosa L., Annonaceae) seed germination: morphological and anatomical changes. *Agron.ía Colombiana* **31**, 176–183 (2013).

[5] Ahmed, R. H. A. & Mariod, A. A. Annona squamosa: phytochemical constituents, bioactive compounds, traditional and medicinal uses. *Wild Fruits: Composition, Nutritional Value and Products*, 143–155 (2019).

[6] Ma, C.-y, Chen, Y., Chen, J., Li, X. & Chen, Y. A Review on Annona squamosa L.: Phytochemicals and Biological Activities. *Am. J. Chin. Med.* **45**, 1–32 (2017). 28659034 10.1142/S0192415X17500501

[7] Pellicciari, C., Biggiogera, M. & Pellicciari. *Histochemistry of single molecules* (Springer, 2017).

[8] Kumar, M. et al. Custard apple (Annona squamosa L.) leaves: Nutritional composition, phytochemical profile, and health-promoting biological activities. *Biomolecules* **11**, 614 (2021). 33919068 10.3390/biom11050614 PMC8143160

[9] Alali, F. Q., Liu, X.-X. & McLaughlin, J. L. Annonaceous acetogenins: recent progress. *J. Nat. Prod.* **62**, 504–540 (1999). 10096871 10.1021/np980406d

[10] Zafra-Polo, M. C., Figadère, B., Gallardo, T., Tormo, J. & Cortes, D. Natural acetogenins from Annonaceae, synthesis and mechanisms of action. *Phytochemistry* **48**, 1087–1117 (1998).

[11] Liaw, C.-C., Liou, J.-R., Wu, T.-Y., Chang, F.-R. & Wu, Y.-C. Acetogenins from annonaceae. *Prog. Chem. Org. Nat. Prod.* **101**, 113–230 (2016). 10.1007/978-3-319-22692-7_2 26659109

[12] Bhardwaj, R., Pareek, S., Sagar, N. & Vyas, N. Bioactive compounds of Annona. *Bioactive compounds in underutilized fruits and nuts*, 37–62 (2020).

[13] Mohammed, M. A. et al. Comprehensive Tools of Alkaloid/Volatile Compounds–Metabolomics and DNA Profiles: Bioassay-Role-Guided Differentiation Process of Six Annona sp. Grown in Egypt as Anticancer Therapy. *Pharmaceuticals* **17**, 103 (2024). 38256936 10.3390/ph17010103 PMC10821326

[14] Oo, W. M. & Khine, M. M. Pharmacological Activities of Annona squamosa: Updated. *Chemistry* **3**, 86–93 (2017).

[15] Pardhasaradhi, B., Reddy, M., Ali, A. M., Kumari, A. L. & Khar, A. Differential cytotoxic effects of Annona squamosa seed extracts on human tumour cell lines: role of reactive oxygen species and glutathione. *J. Biosci.* **30**, 237–244 (2005). 15886460 10.1007/BF02703704

[16] Al-Nemari, R. et al. GC-MS profiling and assessment of antioxidant, antibacterial, and anticancer properties of extracts of Annona squamosa L. leaves. *BMC Complement. Med. Ther.* **20**, 1–14 (2020). 33023568 10.1186/s12906-020-03029-9 PMC7541211

[17] Joy, B. & Remani, P. Antitumor constituents from Annona squamosa fruit pericarp. *Med. Chem. Res.* **17**, 345–355 (2008).

[18] Suresh, K., Manoharan, S., Panjamurthy, K. & Kavitha, K. Chemopreventive and antilipidperoxidative efficacy of Annona squamosa bark extracts in experimental oral carcinogenesis. *Pak. J. Biol. Sci.* **9**, 2600–2605 (2006).

[19] Manoharan, J. P., Palanisamy, H. & Vidyalakshmi, S. Overcoming multi drug resistance mediated by ABC transporters by a novel acetogenin-annonacin from Annona muricata L. *J. Ethnopharmacol.* **322**, 117598 (2024). 38113989 10.1016/j.jep.2023.117598

[20] Sharma, Y., Arora, M. & Bala, K. The potential of immunomodulators in shaping the future of healthcare. *Discov. Med.* **1**, 37 (2024).

[21] Tu, W. et al. Isolation, characterization and bioactivities of a new polysaccharide from Annona squamosa and its sulfated derivative. *Carbohydr. Polym.* **152**, 287–296 (2016). 27516275 10.1016/j.carbpol.2016.07.012

[22] Haggag, W. M. & Nofal, M. Improving the biological control of Botryodiplodia disease on some Annona cultivars using single or multi-bioagents in Egypt. *Biol. Control* **38**, 341–349 (2006).

[23] Abdelkawy, M. Identification of Three Egyptian Annona Cultivars Morhologically and Biochimically using Rapd Analysis. *Alex. J. Agric. Sci.* **62**, 415–421 (2017).

[24] Habib, A.-A. M. False-positive alkaloid reactions. *J. Pharm. Sci.* **69**, 37–43 (1980). 7354438 10.1002/jps.2600690111

[25] Ma, C. et al. Three new cytotoxic annonaceous acetogenins from the seeds of Annona squamosa. *Nat. Prod. Res.* **38**, 1135–1139 (2024). 36260488 10.1080/14786419.2022.2134362

[26] Chen, Y., Chen, J.-w. Wang, Y., Xu, S.-s & Li, X. Six cytotoxic annonaceous acetogenins from Annona squamosa seeds. *Food Chem.* **135**, 960–966 (2012). 22953811 10.1016/j.foodchem.2012.05.041

[27] Nandhakumar, E. & Indumathi, P. In vitro antioxidant activities of methanol and aqueous extract of Annona squamosa (L.) fruit pulp. *J. Acupunct. Meridian Stud.* **6**, 142–148 (2013). 23787283 10.1016/j.jams.2012.09.002

[28] Méndez-Chávez, M., Ledesma-Escobar, C. A., Hidalgo-Morales, M., Rodríguez-Jimenes, G. D. C. & Robles-Olvera, V. J. Antifungal activity screening of fractions from Annona cherimola Mill. leaf extract against Fusarium oxysporum. *Arch. Microbiol.* **204**, 330 (2022). 35579717 10.1007/s00203-022-02944-4

[29] Nonfon, M., Lieb, F., Moeschler, H. & Wendisch, D. Four annonins from Annona squamosa. *Phytochemistry* **29**, 1951–1954 (1990).

[30] Renzhou, Y., Xiangci, Z., Shujun, W. & Guowei, Q. Annonsilin Aa novel seco-tris-tetrahydrofuranyl annonaceous acetogenin. *Acta Botanica Sin.* **37**, 492–495 (1995).

[31] LI, Y. Two new annonaceous acetogenins from seeds of Annona squamosa. *Chin. Trad. Herb Drug.* **24**, 2375–2381 (2017).

[32] Cheng-Yao, M., Jia-Hui, L., Xiang, L., Xiao, L. & Jian-Wei, C. Eight new cytotoxic annonaceous acetogenins from the seeds of Annona squamosa. *Chin. J. Nat. Med.* **17**, 291–297 (2019). 31076132 10.1016/S1875-5364(19)30032-9

[33] Hopp, D. C., Alali, F. Q., Gu, Z.-m & McLaughlin, J. L. Three new bioactive bis-adjacent THF-ring acetogenins from the bark of Annona squamosa. *Bioorg. Med. Chem.* **6**, 569–575 (1998). 9629470 10.1016/s0968-0896(98)00018-2

[34] Sahai, M. et al. Annonaceous acetogenins from the seeds of Annona squamosa. Adjacent bis-tetrahydrofuranic acetogenins. *Chem. Pharm. Bull.* **42**, 1163–1174 (1994).

[35] Li, X., Chen, X.-L., Chen, J.-W. & Sun, D.-D. Annonaceous acetogenins from the seeds of Annona squamosa. *Chem. Nat. Compd.* **46**, 101–105 (2010).

[36] Miao, Y. et al. Four cytotoxic annonaceous acetogenins from the seeds of Annona squamosa. *Nat. Prod. Res.* **30**, 1273–1279 (2016). 26181648 10.1080/14786419.2015.1055490

[37] Miao, Y.-J. et al. Three cytotoxic Annonaceous acetogenins from the seeds of Annona squamosa. *Phytochem. Lett.* **16**, 92–96 (2016). 10.1080/14786419.2015.1055490 26181648

[38] Fujimoto, Y. et al. Annonaceous acetogenins from the seeds of Annona squamosa. Non-adjacent bis-tetrahydrofuranic acetogenins. *Chem. Pharm. Bull.* **42**, 1175–1184 (1994).

[39] Hopp, D. C., Zeng, L., Gu, Z.-m & McLaughlin, J. L. Squamotacin: an annonaceous acetogenin with cytotoxic selectivity for the human prostate tumor cell line (PC-3). *J. Nat. Prod.* **59**, 97–99 (1996). 8991957 10.1021/np960124i

[40] Guideline, I. H. T. Validation of analytical procedures: text and methodology. *Q2 (R1)* **1**, 05 (2005).

[41] Weyh, C., Krüger, K., Peeling, P. & Castell, L. The role of minerals in the optimal functioning of the immune system. *Nutrients* **14**, 644 (2022). 35277003 10.3390/nu14030644 PMC8840645

[42] Tam, M., Gomez, S., Gonzalez-Gross, M. & Marcos, A. Possible roles of magnesium on the immune system. *Eur. J. Clin. Nutr.* **57**, 1193–1197 (2003). 14506478 10.1038/sj.ejcn.1601689

[43] Omofuma, O. O. et al. Trends in reported calcium and magnesium intake from diet and supplements by demographic factors: National Health and Nutrition Examination Survey, 2003–2018. *J. Acad. Nutr. Dietetics* **124**, 1288–1301.e1285 (2024). 10.1016/j.jand.2024.04.017 PMC12244524 38718857

[44] Shehata, M. G., Abu-Serie, M. M., Abd El-Aziz, N. M. & El-Sohaimy, S. A. Nutritional, phytochemical, and in vitro anticancer potential of sugar apple (Annona squamosa) fruits. *Sci. Rep.* **11**, 6224 (2021). 33737634 10.1038/s41598-021-85772-8 PMC7973736

[45] das Chagas Lima, N. N., Faustino, D. C., Allahdadi, K. J., de Aragão França, L. S. & Pinto, L. C. Acetogenins from Annonaceae plants: potent antitumor and neurotoxic compounds. *PharmaNutrition* **20**, 100295 (2022).

[46] Ma, C. et al. Three new antitumor annonaceous acetogenins from the seeds of Annona squamosa. *Nat. Prod. Res.* **31**, 2085–2090 (2017). 28064519 10.1080/14786419.2016.1274897

[47] Jacobo-Herrera, N. et al. Selective acetogenins and their potential as anticancer agents. *Front. Pharmacol.* **10**, 783 (2019). 31379567 10.3389/fphar.2019.00783 PMC6657400

[48] Bala, S., Nigam, V., Singh, S. S., Kumar, A. & Kumar, S. Evaluation of nutraceutical applications of Annona squamosa L. Based Food Products. *J. Pharmacogn. Phytochem.* **7**, 827–831 (2018).

[49] Souza, F. T. C. et al. Production of nutritious flour from residue custard apple (Annona squamosa L.) for the development of new products. *J. Food Qual.* **2018**, 5281035 (2018).

[50] Huang, C. et al. A novel heteropolysaccharide isolated from custard apple pulp and its immunomodulatory activity in mouse macrophages and dendritic cells. *Heliyon* **9**, e18521 (2023). 37554813 10.1016/j.heliyon.2023.e18521 PMC10404978

[51] Kritchevsky, D., Martak, D. S. & Rothblat, G. H. Detection of bile acids in thin-layer chromatography. *Anal. Biochem.* **5**, 388–392 (1963). 14035852 10.1016/0003-2697(63)90040-x

[52] Jia, Z. & Tian, C. Quantitative determination of polyethylene glycol with modified Dragendorff reagent method. *Desalination* **247**, 423–429 (2009).

[53] Sandeep, W. et al. Evaluation of phytochemical & antimitotic potential of annona reticulata extracts by onion root model. *Chem. Proc.* **3**, 137 (2020).

[54] Tolosa, L., Donato, M. T. & Gómez-Lechón, M. J. In *Protocols in In vitro hepatocyte research* (Springer, 2015).

[55] Sherma, J. & Fried, B. In *Journal of Chromatography Library* Vol. 38 (ed Brian A. Bidlingmeyer) 105–127 (Elsevier, 1987).

[56] Melchart, D., Linde, K., Worku, F., Bauer, R. & Wagner, H. Immunomodulation with Echinacea—a systematic review of controlled clinical trials. *Phytomedicine* **1**, 245–254 (1994). 23195946 10.1016/S0944-7113(11)80072-3

[57] Quero-Jiménez, P. Total carbohydrates concentration evaluation in products of microbial origin. *Afinidad* **76**, 195–203 (2019).

[58] Jiménez, M. D. A., Gil, M. I. S., Corvillo, M. A. P. & Díez, L. M. P. Determination of calcium and magnesium in milk by complexometric titration using protein precipitation and complexation with Palladiazo or other indicators. *Analyst* **113**, 633–635 (1988). 3407961 10.1039/an9881300633

[59] Liyanage, J. A. & Janaratne, T. Chemical speciation: a guide to understand titrimetric analysis. *J. Chem. Educ.* **79**, 635 (2002).

[60] Wilkinson, R. H. A micro-method for serum calcium and serum magnesium. *J. Clin. Pathol.* **10**, 126–135 (1957). 13428869 10.1136/jcp.10.2.126 PMC1024024

[61] Larranaga, N. & Hormaza, J. I. DNA barcoding of perennial fruit tree species of agronomic interest in the genus Annona (Annonaceae). *Front. Plant Sci.* **6**, 589 (2015). 26284104 10.3389/fpls.2015.00589 PMC4519677

